# Diurnal sheltering preferences and associated conservation management for the endangered sandhill dunnart, *Sminthopsis psammophila*

**DOI:** 10.1093/jmammal/gyab024

**Published:** 2021-04-23

**Authors:** Joanna Riley, Jeff M Turpin, Matt R K Zeale, Brynne Jayatilaka, Gareth Jones

**Affiliations:** 1 School of Biological Sciences, University of Bristol, Bristol, United Kingdom; 2 School of Environmental and Rural Science, University of New England, Armidale, New South Wales, Australia; 3 APA Group, Spring Hill, Queensland, Australia

**Keywords:** Australia, conservation management, Great Victoria Desert, habitat preferences, radiotracking, sandhill dunnart, shelter, *Sminthopsis psammophila*, spatial ecology, threatened species

## Abstract

Dasyurids are small mammals that can conserve energy and water by using shelters that insulate against extreme conditions, prevent predation, and facilitate torpor. To quantify the diurnal sheltering requirements of a poorly known, endangered dasyurid, the sandhill dunnart, *Sminthopsis psammophila*, we radiotracked 40 individuals in the Western Australian Great Victoria Desert between 2015 and 2019. We assessed the effect of habitat class (broad habitat features), plot-level (the area surrounding each shelter), and shelter characteristics (e.g., daily temperature ranges), on shelter selection and sheltering habitat preferences. Two hundred and eleven diurnal shelters (mean of 5 ± 3 shelters per individual) were located on 363 shelter days (the number of days each shelter was used), within mature vegetation (mean seral age of 32 ± 12 years postfire). Burrows were used on 77% of shelter days and were typically concealed under mature spinifex, *Triodia* spp., with stable temperature ranges and northern aspects facing the sun. While many burrows were reused (*n* = 40 across 175 shelter days), spinifex hummock shelters typically were used for one shelter day and were not insulative against extreme temperatures. However, shallow scrapes within *Lepidobolus deserti* hummock shelters had thermal advantages and log shelters retained heat and were selected on cooler days. *Sminthopsis psammophila* requires long-unburned sheltering habitat with mature vegetation. Summer fires in the Great Victoria Desert can be extensive and destroy large areas of land, rendering them a key threat to the species. We conclude that the survey and conservation of *S. psammophila* requires attention to long-unburned, dense lower stratum swale, sand plain, and dune slope habitats, and the tendency of *S. psammophila* to burrow allows the species to survive within the extreme conditions of its desert environment.

Deserts occupy one-third of the Earth’s land surface and in Australia, the arid zone covers approximately 70% of the continent (Laity 2009; Beard et al. 2014). Australian deserts are defined by aridity, irregular rainfall, extreme temperatures, and nutrient-poor soils (Masters 1993; Allan and Southgate 2002; Holmgren et al. 2006). Despite hostile environmental conditions, the Australian arid zone supports a diverse array of small mammals and approximately one-half of the continent’s carnivorous marsupials, the dasyurids (Dickman 2003). The high diversity of dasyurids across arid Australia has been linked to several specialized traits enabling survival (Friend 1993; Dickman 2003; Waudby and Petit 2017). For example, the ability of dasyurids to enter daily torpor (a controlled reduction in body temperature and basal metabolic rate—Geiser and Ruf 1995) is critical for the conservation of energy and water, facilitates breeding, and appears to prolong life span, allowing for survival in drought- and fire-prone ecosystems (Geiser 2004). Intrinsically linked to torpor is shelter type and use, as different shelter microclimates and substrates assist torpor and appear essential for physiological regulation (Körtner et al. 2008; Warnecke et al. 2008). Most desert dasyurids are solitary, display a low fidelity to shelter sites, and use a mobile life strategy to track shifting resources in dynamic desert environments (Haythornthwaite and Dickman 2006; Waudby and Petit 2017; Baker and Dickman 2018).

Globally, many small desert mammal species shelter within burrows, for example, kangaroo rats (*Dipodomys* spp.), jerboas (*Allactaga* spp.), and hopping mice (*Notomys* spp.) (Kinlaw 1999; van Dyck and Strahan 2008). Burrows form vital micro-refuges, particularly where alternative, thermally suitable refuges are rare, that provide protection from climatic extremes, predation, and fire and are linked to physiology, movement, and diet (Kinlaw 1999; Baker and Dickman 2018). While burrows have been shown to buffer against temperature extremes (Kinlaw 1999; Degen 2012), the thermal diffusivity of sandy soils, combined with shallow burrow depths, require some burrowing desert species to frequently utilize torpor and passive rewarming, e.g., sun basking, to survive (Lovegrove et al. 1999; Körtner et al. 2008; Pavey and Geiser 2008). Burrows also provide antipredation benefits that lower the risk of predation for small arid zone mammals worldwide (Reichman and Smith 1990; Kinlaw 1999; Laundré et al. 2001; Bleicher and Dickman 2020).

Burrowing is less commonly reported for *Sminthopsis* spp. (dunnarts) and only a few dunnart species are known to excavate their own burrows. Most *Sminthopsis* spp. shelter under or within logs, woody debris, or dense vegetation (e.g., *S. dolichura*—Morton 1978) or use soil-crevices (e.g., *S. crassicaudata*, *S. macroura*, and *S. douglasi*—Waudby and Petit 2017; Woolley 2017). *Sminthopsis youngsoni* typically usurps the burrows of other taxa, usually spiders, scorpions, or rodents (Haythornthwaite and Dickman 2006; Baker and Dickman 2018), whereas *S. hirtipes* usurps burrows of spinifex hopping mouse (*N. alexis*) and central netted dragon (*Ctenophorus nuchalis*—Dickman *et al.* 1993). Overall, there are 12 species of arid zone *Sminthopsis* but the role that shelter plays in physiologically sustaining these populations is poorly known. Given the high rate of mammalian decline in Australia’s arid zone, coupled with the warming of Australian deserts (Hughes 2003; Steffen et al. 2009; IPCC 2014; CSIRO 2017), understanding habitat and shelter requirements is vital to dasyurid conservation, particularly to ameliorate threatening processes such as predation and wildfire (Burbidge et al. 1989; McKenzie et al. 2007; Woinarski et al. 2015).

The sandhill dunnart, *Sminthopsis psammophila*, is listed as endangered nationally in Australia under the Environment Protection and Biodiversity Conservation Act of 1999. It is a semiarid specialist that has declined significantly in range, persisting only in three widely separated populations (Fig. 1). Key threats include predation and increasingly frequent droughts and wildfires (Doherty et al. 2015; Dutta et al. 2016; Woinarski and Burbidge 2016; Murphy et al. 2019). Empirical studies are lacking, and the species historically has been difficult to detect, particularly in Western Australia (Burbidge et al. 1976; Pearson and Robinson 1990; Brennan et al. 2012; Gaikhorst and Lambert 2014). In South Australia, *S. psammophila* has been associated with flammable spinifex (*Triodia* spp.) hummock grassland habitats that provide spikey and neurotoxic micro-refuges which reduce perceived predation risk (Churchill 2001a; Laundré et al. 2001). Limited radiotracking data indicated shelters were associated with large, mature “Stage 3” spinifex hummocks (Fig. 2), although these hummocks constituted only 5% of the locally available hummocks (Churchill 2001a). Other studies have emphasized the significance of *Triodia* spp. hummocks > 40 cm high and complex habitats with an abundance of logs (McLean 2015; Moseby et al. 2016). However, burrow use for *S. psammophila* has been overlooked. As the thermal biology and physiology of *S. psammophila* resembles other sympatric dasyurids including species of dunnart (Withers and Cooper 2009), we hypothesize that *S. psammophila* may display similar adaptations to its arid environment, including diurnal burrow use to conserve energy and water and reduce predation risk.

**Fig. 1.**
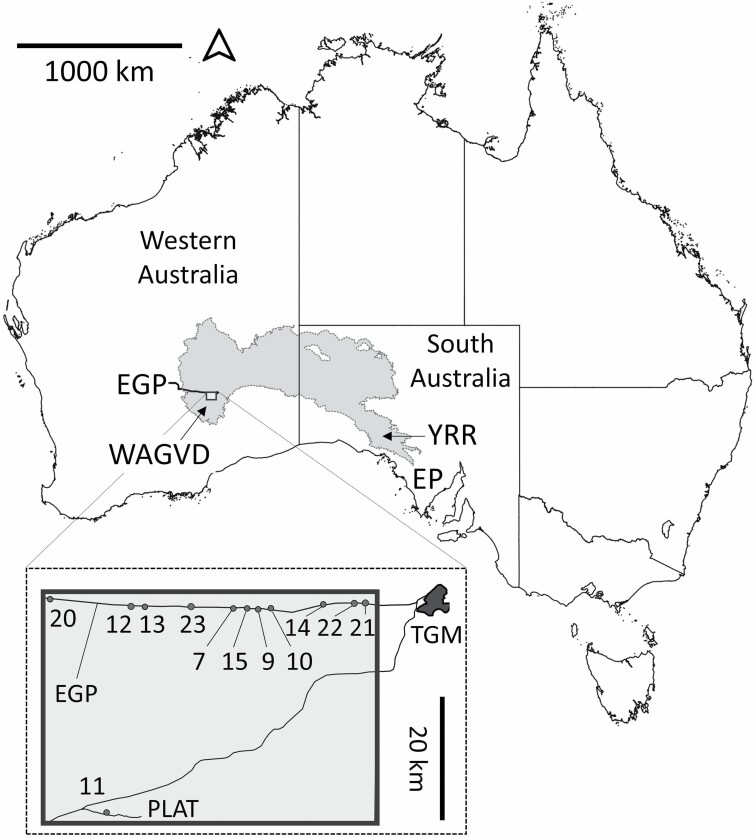
*Sminthopsis psammophila* distribution map with the Great Victoria Desert (GVD; gray) bioregion indicated. Known *S. psammophila* populations are located in the Western Australian Great Victoria Desert (WAGVD), within or near the Yellabinna Regional Reserve (YRR), and on Eyre Peninsula (EP). Inset: individuals (*n =* 40) were radiotracked at 11 sites along the APA Group Eastern Goldfields Pipeline (EGP) and Plumridge Lakes Access Track (PLAT) near Tropicana Gold Mine (TGM) between 2015 and 2019. See Supplementary Data SD1 for site descriptions.

**Fig. 2.**
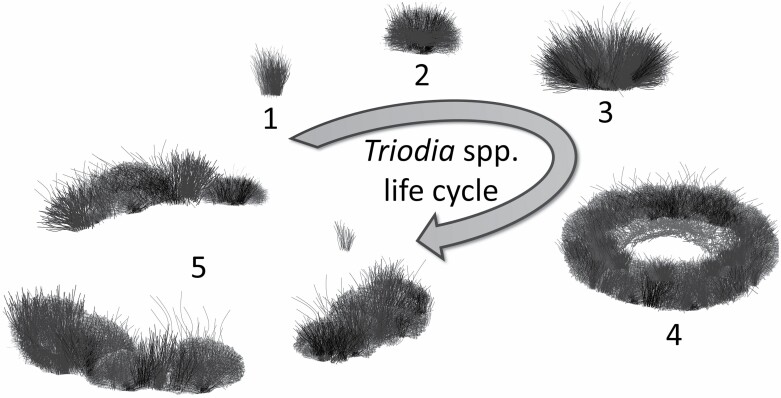
Ring-forming spinifex (*Triodia* spp.) life stages (1–5; redrawn from Churchill 2001b). Stage 1—new growth hummock, not suitable habitat for *Sminthopsis psammophila*; Stage 2—hummock too small and dense for *S. psammophila*; Stage 3—becomes suitable for *S. psammophila*; Stage 4—hummock opens into ring with soft, dead leaves centrally (burrows were often concealed underneath); Stage 5—hummock breaks apart and continues spreading, larger sections remain suitable for sheltering.

Our study addresses knowledge gaps in the conservation biology of *S. psammophila*. We evaluated whether: 1) shelters are selected within specific habitat classes; 2) certain shelter types and habitat features are preferred by *S. psammophila*; 3) shelter preferences differ with sex, reproductive status, or both; and 4) shelter selection is associated with the thermal effect (temperature range) of shelter types. We also predicted that shelter selection may differ across populations of *S. psammophila*. We then used our data to suggest improvements for the survey and conservation management of *S. psammophila* throughout its geographic range.

## Materials and Methods

### Study area

Our research was conducted largely along the Eastern Goldfields Pipeline (EGP) at 11 sites up to 60 km west of Tropicana Gold Mine (TGM), located at 29°14′55″S, 124°33′21″E, and at one site 60 km south of TGM (Fig. 1; Supplementary Data SD1) in the Western Australian Great Victoria Desert (WAGVD). The WAGVD study site is classified as “Plains and dunes (longitudinal and ring dunes) with interdune corridors and plains; occasional salt pans” at an average (± *SD*) elevation of 402 ± 22 m a.s.l. (DAFWA 2014) and is dominated by *T. desertorum* and *T. basedowii* hummock grasslands with scattered eucalypts including marble gums (*E. gongylocarpa*) situated over wattle scrub (*Acacia* spp.) and mallee (*Eucalyptus* spp.—Beard et al. 2014). The study site soils are “Yellow deep sand, Soil Group 446” except for “Mulga” habitat class soils where a variable depth sandy loamy clay crust is present (Schoknecht and Pathan 2013). Ambient temperature (*T*_A_) and weather data were recorded by the TGM weather station during the study. Mean maximum (*T*_A_.max.r) and minimum (*T*_A_.min.r) ambient temperature ranges were calculated for both reproductive seasons. Annual long-term weather data of mean maximum temperature (*T*_LT_.max), mean minimum temperature (*T*_LT_.min), highest temperature (*T*_LT_.hi), and lowest temperature (*T*_LT_.lo) were compared from the nearest available Bureau of Meteorology weather stations with long-term data sets to the study site and the two other *S. psammophila* populations (BOM 2020).

### Trapping, tagging, and handling

Trapping, radiotransmitter attachment (tagging), and handling procedures followed Turpin and Riley (2017) and conformed to ASM guidelines (Sikes et al. 2016). Approvals were obtained from the University of Bristol and the Department of Biodiversity, Conservation and Attractions (DBCA) ethical review committees under DBCA license 08-001295-4. Trapping was performed biannually between 2015 and 2019 in the reproductive season (September and October) and the nonreproductive season (March and April). Individuals were captured in 65-cm deep by 30-cm diameter polyvinyl chloride (PVC) pitfall traps (Read et al. 2015) and grouped by sex (male = M; female = F) and reproductive status (reproductive = R; nonreproductive = nR). Trapping effort (trap-nights) was calculated by multiplying the number of pitfall traps open by the number of nights open. Trap sites were closed once animals were tagged. Crown length (mm), short and long pes (mm), body mass (g), tail length (mm), and testes width and length (mm) or pouch condition (undeveloped, used, virgin) were measured to assess age, reproductive status, and health. Lightweight radiotransmitters (“Pip series”; 0.2–0.4 g; Biotrack Ltd., Wareham, Dorset, United Kingdom) weighing less than 5% of an individual’s body mass were attached to the lower dorsal area with cold-curing tissue adhesive (Vetbond) after clipping a small area of the fur (Kenward 1987). Females with large pouch young were not tagged. Individuals were released at sunset then radiotracked until midnight to determine their core sheltering area using ATS R410 scanning receivers and 3-element folding Yagi directional antennas (Advanced Telemetry Systems Australia, Queensland, Australia). Individuals were tracked until radiotransmitters naturally detached after 1–48 days. Shelter locations were recorded every morning after tagging using a global positioning system (GPS) device (Garmin, eTrex, 5–15 m accuracy; Garmin Europe Ltd., Romsey, United Kingdom) more than 30 min after sunrise to avoid disturbance. All further data on shelters, such as type or characteristics, were collected after radiotransmitters detached.

### Site level

Using satellite imagery in QGIS (QGIS 2021, Open Source Geospatial Foundation Project, www.qgis.osgeo.org), we generated habitat maps for the home range areas of all radiotracked individuals using seven habitat classes (Table 1) and determined the mean ± *SD* minimum fire age (the number of years since the last wildfire) in each case. Digitized habitat maps were ground-validated with field surveys performed during radiotracking. The number of days each shelter was used (shelter days) and the number of days a shelter was used before moving to a new shelter were recorded.

**Table 1.** Habitat class descriptions and the proportions available in the study site were used to determine the effect of habitat class on shelter selection by *Sminthopsis psammophila* in the Western Australian Great Victoria Desert (WAGVD).

Habitat class (proportion available)	Description
Swale or sand plain (0.23)	Hummock grasslands: the lower stratum is dominated by spinifex (*Triodia* spp.) of a height of up to 75 cm with a dense and varied middle stratum and little to no upper stratum. Sparse to no litter or logs. Gradient = 0°.
Crest (0.04)	Sand dune apexes dominated by fine, loose yellow sandy terrain and patchy open vegetation. Where present, vegetation is usually intermittent (widely spaced) spinifex with an open middle stratum, a sparse upper stratum, and sparse litter or logs.
North slope (0.11)	Sloping habitat on the northern aspect of sand dunes, excluding the dune crest, with a steep gradient near crest becoming gentle then terminating where slope = 0°. Vegetation is noticeably denser than dune crest; lower stratum is dominated by spinifex of a height of up to 75 cm with a dense and varied middle stratum and sparse upper stratum, litter or logs. Usually more gradual and wider than south slope.
South slope (0.09)	Sloping habitat on the southern aspect of sand dunes, excluding the dune crest, with a steep gradient near crest becoming gentle then terminating where slope = 0°. Vegetation is noticeably denser than dune crest; lower stratum is dominated by spinifex of a height of up to 75 cm with a dense and varied middle stratum and sparse upper stratum, litter or logs. Usually steeper and not as wide as north slope.
Woodland (0.30)	Open woodland dominated by marble gums (*Eucalyptus gonglyocarpa*), *Callitris* sp., *Allocasuarina* spp., *Acacia* spp. (but excludes *Acacia aneura* complex “Mulga” habitat). Includes dense thickets of mallee (*Eucalyptus* spp.) woodlands. Upper stratum is > 2 m tall with a variable, dense middle stratum, sparse lower stratum. Ground level is dominated by litter or logs.
Mulga (0.21)	*Acacia aneura* complex woodland typically in sandy loam soil with a varied and dense middle stratum, none to a very low proportion of cover in the lower stratum with a high proportion of litter or logs. Typically has a high proportion of fallen large dead mulga trunks, logs, and branches at ground level. Noticeably different and lacks spinifex.
Burned (0.03)	Recently burned (within the past 2 years) habitat. No old growth lower, middle, or upper stratum vegetation remains but limited new growth may have developed. Dominated by bare ground and burned woody debris. Typically, no spinifex or lower stratum.

### Plot level

We investigated the shelter use of *S. psammophila* at the plot level by using 25-m^2^ shelter plots (*n* = 211) surrounding each centrally located shelter. Shelter plots were compared with an equal number of paired, randomly selected 25-m^2^ plots (*n* = 211) assigned in QGIS using the “Random Points” tool within individual home range areas. Plot characteristics measured in the field were floristic richness (sum of living species per plot), dominant *Triodia* spp. (either *T. desertorum*, *T. basedowii*, or *T.* sp. *rigidissima*), hummock life stage from “Stage 1” to “Stage 5” (Fig. 2), terrain slope (°) measured using an inclinometer application (Clinometer; Stephanskirchen, Germany; www.plaincode.com), and terrain aspect (either north-facing, south-facing, or no aspect). We calculated elevation (m a.s.l.) and the distances (m) of shelters to the nearest dune crest (dist.Crest) in QGIS to examine if shelters were selected at particular elevations and whether the conservation of dune crests alone is sufficient to protect *S. psammophila*. To investigate the structural density of habitat surrounding shelters, the proportion of ground level, lower, and middle, strata within shelter and random plots were compared. Structural habitat strata were classified as follows: 1) Ground = the sum of the proportions of sand, litter, logs, *Triodia* spp., and other vegetation (e.g., sedge or native grass) within the plot at a height of < 0.15 m; 2) Lower = the sum of the proportions of litter, logs, *Triodia* spp., and other vegetation within the plot of height between 0.15 and 0.75 m; 3) Middle = the sum of the proportion of vegetation (e.g., shrubs or trees) within the plot of height between 0.75 and 2 m.

### Shelter level

At the shelter level, characteristics of individual shelters were compared with the nearest suitable shelter of the same type at an equal number of random points assigned in QGIS within individual home ranges. Not all shelters were measured as some were occupied or inaccessible. The number of randomly selected log shelters (*n* = 18) was tripled to improve statistical power. Only random shelters that were equal to or larger in size than the smallest shelter of its type recorded in this study were compared and shelters that were much larger than a plausible shelter for *S. psammophila* were not compared. The *Schoenus hexandrus* (*n* = 1) shelter, the bark shelter (*n* = 1), and the mallee stump (*n* = 1) hollow were excluded from this analysis due to low sample sizes. Characteristics of the shelters and the vegetation that covered each shelter, when present, were recorded. Burrows were defined as subterranean excavations > 8 cm deep that fully concealed an individual. Burrow dimensions recorded were entrance width, height, and depth (cm) and entrance aspect (north, south, east, or west). Hummock shelter height (m), greatest width (m), species, and life stage (“Stage 1” to “Stage 5”) were recorded (Fig. 2).

### Temperature

To examine the thermal effect of shelter type [Temperature (T °C)], Thermochron iButton temperature data loggers (iButtons; Model DS1921G, Maxim/Dallas Semiconductor, Dallas, Texas; www.ibutton.com) were deployed to record daily temperature ranges (*T*_r_) within shelters. iButton recordings were checked for accuracy 1 day before deployment using paired hourly recordings by a digital thermometer and deployed after radiotracking in unoccupied shelters verified as vacant using a flashlight in the positions where individuals had sheltered; all shelter types were measured excluding the piece of bark that was occupied. Control iButtons were deployed at ground level to record control ground temperature ranges (GT_r_). Maximum (GT_max_) and minimum (GT_min_) control ground temperatures during the study were recorded to measure the thermal extremes of WAGVD temperatures at ground level. Replicated tests using iButtons recorded the mean temperatures of all habitat classes (HT_x_), excluding “Woodland” and “Burned” habitats that were used infrequently during the study, to determine whether HT_x_ varied among habitat classes. iButtons were deployed within similar arboreal shade levels.

### Statistical analysis

Statistical methods at site level used a chi-square analysis with Yate’s correction, as “Crest” habitats had an expected value (*n* = 8 shelters) that was < 5% of total observations, to examine if individuals favored shelters within a particular habitat class. We examined whether the observed number of shelters recorded in each habitat class divided by the total number of shelters located in the study site departed from the expected number of shelters if distributed proportionally to the area of the corresponding habitat class available. To quantify this, the proportion available of each habitat class was calculated by dividing the sum area of each habitat class by the overall study site area (Table 1). “Burned” habitat was excluded from the chi-square analysis because of its infrequent use (2% of the total study site, used for one shelter day only). The *Z*-statistic was used to calculate Bonferroni’s confidence intervals (Neu *et al.* 1974; Byers *et al.* 1984) and to establish whether individuals positively or negatively selected a habitat class.

To examine whether shelter plot features differed significantly from those of paired random plots, we first performed univariate analyses on the variables (paired *t*-tests with Bonferroni corrections for normally distributed data, Wilcoxon rank sums tests for nonparametric data, and chi-square tests for categorical data—Carr et al. 2018). To determine which response variables contributed most to explaining the variation among shelter and random plots, we performed a series of generalized linear mixed-effects models (GLMMs) using maximum likelihood estimations, a binomial distribution and logit link function using the glmmTMB package (Brooks et al. 2017). The sampling units were plots. Plot type (shelter or random) was a fixed effect. The individual and site were included as random effects to account for pseudoreplication (Bolker et al. 2009). Spearman’s correlation tests assessed variables prior to modeling to determine multicollinearity (|*r*| > 0.6 coefficient threshold). When correlation was found, the variable with the least explanatory power was removed to simplify the model. Data were standardized using mean and standard deviation ((*x* − μ)/σ) to provide useful comparisons of effect size. Akaike’s information criterion scores for small sample sizes (AICc) identified the most parsimonious model that explained the most amount of variance. Pseudo-*R*^2^ (1 − (residual deviance/null deviance)) were applied to explain the fit of each model and a final set of the best-fitting models were chosen using delta AICc (Δ*i* ≤ 2). We applied model averaging on the final best models to increase precision in the calculation of estimates and associated *SE* (Burnham and Anderson 2002). To examine whether shelter plot variables differed significantly with sex or reproductive status we used a two-way ANOVA with post hoc Tukey tests.

At the shelter level, we compared the characteristics of shelters with those of random corresponding shelters using univariate tests (paired *t*-tests with Bonferroni corrections for normally distributed data, Wilcoxon rank sum tests for nonparametric data, or chi-square tests for categorical data). We used a one-way ANOVA with a post hoc Tukey test to determine whether shelter type had a significant effect on the number of days it was used (shelter days).

We examined the effect of temperature (T °C) on shelter selection by comparing daily shelter *T*_r_ with control GT_r_ using paired *t*-tests with Bonferroni corrections. The bark shelter was occupied and not measured, and the *S. hexandrus* and mallee stump *T*_r_ were excluded from further analyses due to small sample sizes. A one-way ANOVA and post hoc Tukey test examined if there was a significant effect of mean maximum daily recorded temperature (*T*_max_) on the type of shelter selected. A one-way ANOVA and post hoc Tukey test examined the effect of habitat class on mean ± *SD* daily temperature (HT_x_). Weather data were supplied by the weather station at TGM. All statistical analyses were performed in R 3.5.1 and RStudio 1.1.463 (R Core Team and RStudio Team 2018). In all tests, significance was set at *P* < 0.05. Normally distributed variables are given as mean ± *SD* and non-normal data as median ± *IQR*.

## Results

Forty *S. psammophila* were captured in 7,200 trap-nights (RM = 15, nRM = 10, RF = 7, nRF = 8). In total, 211 diurnal shelters (mean of 5 ± 3 shelters per individual) were located on 363 shelter days. Shelters were reused on multiple days (mean per individual = 8 ± 9 shelter days). The mean fire age of the study site was 32 ± 12 years (range: 0–40+ years). Detailed site data, such as habitat classes available or minimum fire age range, are given in Supplementary Data SD1. Weather conditions were stable from year to year, excluding a drought (< 50 mm annual rainfall) in 2019 (Supplementary Data SD2). In the reproductive season, *T*_A_.max.r was 26.4–31.7°C and *T*_A_.min.r was 9.5–14.5°C. In the nonreproductive season, *T*_A_.max.r was 29.2–33.1°C and *T*_A_.min.r was 13.9–18.2°C. The maximum *T*_A_ was 47.3°C on 13 January 2019 and minimum *T*_A_ was −2.9°C on 5 July 2017. Long-term annual weather data for all populations is given in Supplementary Data SD3.

**Fig. 3.**
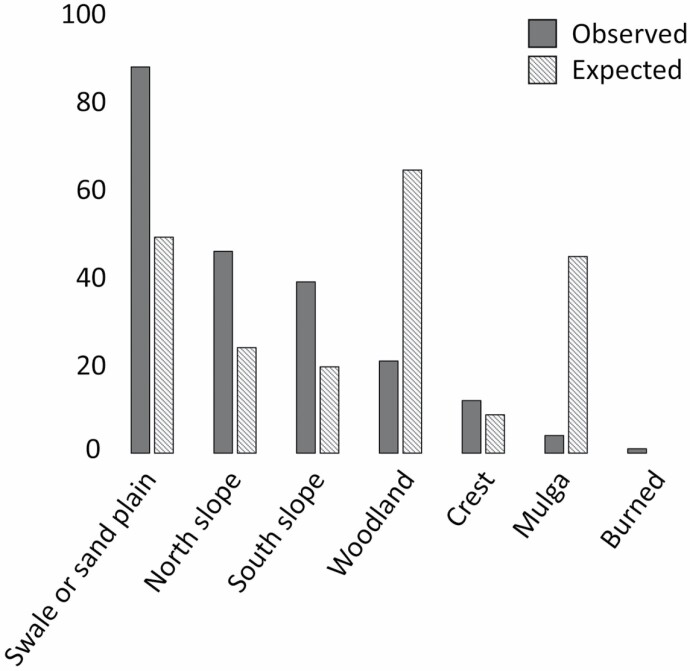
The observed number (*n* = 211) of *Sminthopsis psammophila* shelters (dark gray) within each habitat class of *S. psammophila* in the Western Australian Great Victoria Desert (WAGVD) were compared using a chi-square test with the habitat classes of expected (striped gray) shelters to test if shelters were distributed proportionally to habitat availability (Supplementary Data SD4).

Shelters were not distributed as expected according to the area of each available habitat class (χ ^2^ = 179.8, *d.f.* = 5, *P* < 0.001; Fig. 3). “Swale or sand plain” (*n* = 88 shelters), “North slope” (*n* = 46 shelters), and “South slope” (*n* = 39 shelters) habitats were positively selected, whereas “Woodland” (*n* = 12 shelters) and “Mulga” (*n* = 4 shelters) habitats were avoided and “Crest” habitats (*n* = 12 shelters) were neither positively nor negatively selected (Supplementary Data SD4).

**Fig. 4.**
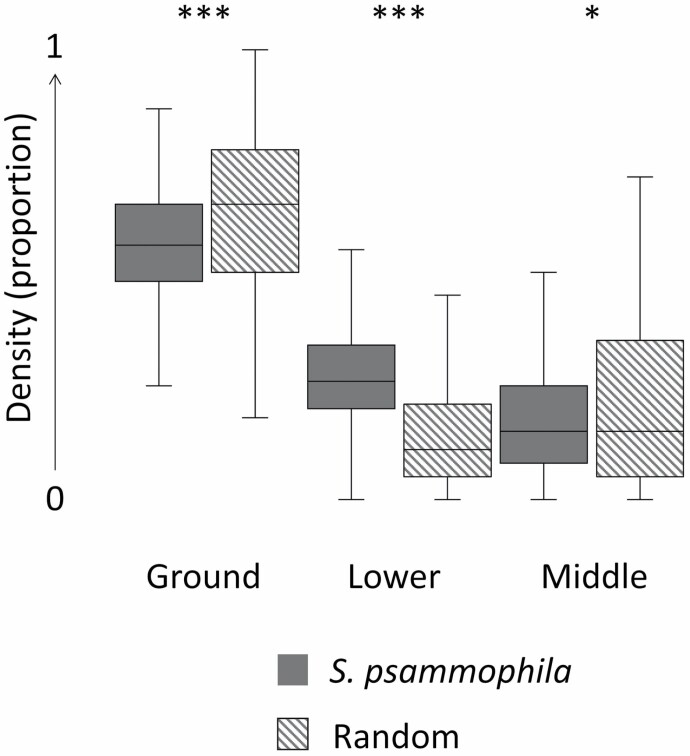
Shelter (dark gray) and random (striped gray) habitat densities within plots (proportion of total plot) at the ground level (Ground), lower stratum (Lower), and middle stratum (Middle) for *Sminthopsis psammophila* in the Western Australian Great Victoria Desert. Significant differences are indicated by *** (*P* < 0.001) and * (*P* < 0.05).

At the plot level, individuals selected shelter plot habitat structures with significantly greater cover at the lower stratum, and significantly less cover at the ground and middle strata compared with random plots (Table 2; Fig. 4). Shelter plots had a significantly higher terrain slope and lower floristic richness compared with random plots (Table 2). Most shelters (*n* = 173) were located within 500 m of a dune crest and were significantly closer to dune crests than random shelters (Table 2). We found no difference for any of the other shelter plot variables measured. *Triodia desertorum* (*n* = 158) was dominant within shelter plots and *T. basedowii* (*n* = 24), *T.* sp. *rigidissima* (*n* = 23), or no spinifex (*n* = 6) were also recorded.

**Table 2.** Habitat variables recorded from shelter and random plots (*n* = 211) and shelter types used by *Sminthopsis psammophila* (*n* = 40) radiotracked in the Western Australian Great Victoria Desert (WAGVD). Normally distributed variables (N) are mean ± *SD*, and non-normally distributed variables (-) are median ± *IQR*. ns = not significant.

Scale	Habitat feature	Shelter	*n*	Random	*n*	Test statistic	*P-*value	Distribution (N = normal)
Plot	Lower	0.27 ± 0.11	211	0.16 ± 0.12	211	*t*_210_ = 9.1	< 0.001	N
	Ground	0.56 ± 0.12	211	0.64 ± 0.18	211	*t*_210_ = −5.1	< 0.001	N
	Middle	0.17 ± 0.13	211	0.21 ± 0.18	211	*t*_210_ = −2.5	< 0.05	N
	Terrain slope (°)	3.9 ± 5.3	211	2.6 ± 4.9	211	*t*_210_ = 2.8	< 0.001	N
	Floristic richness	4.2 ± 1.6	211	4.5 ± 1.7	211	*t*_210_ = −2.0	< 0.05	N
	Dist.Crest (m)	205 ± 287	211	244 ± 316	211	*t*_210_ = −3.8	< 0.001	N
	Elevation (m a.s.l.)	391 ± 17	211	390 ± 14	211	*t*_210_ = 1.0	ns	N
	Dominant *Triodia* spp.	Cat.	211	Cat.	211	χ ^2^ = 12.0, *d.f.* = 9	ns	-
	Dominant *Triodia* stage	Cat.	211	Cat.	211	χ ^2^ = 15.0, *d.f.* = 12	ns	-
	Terrain aspect	Cat.	211	Cat.	211	χ ^2^ = 6.0, *d.f.* = 4	ns	-
Burrow	Entrance height (cm)	4.3 ± 0.9	108	5.7 ± 2	108	*W* = 6,690	< 0.05	-
	Entrance width (cm)	5.2 ± 1.4	108	9.4 ± 3.3	108	*t*_107_ = 12.0	< 0.05	N
	Depth (cm)	37.7 ± 42.6	108	28.4 ± 14.7	108	*W* = 4,467	ns	-
	Aspect	Cat.	108	Cat.	108	χ ^2^ = 27.0, *d.f.* = 3	< 0.001	-
*Triodia* spp.	Species	Cat.	44	Cat.	44	χ ^2^ = 6.0, *d.f.* = 4	ns	-
	Stage	Cat.	44	Cat.	44	χ ^2^ = 15.0, *d.f.* = 12	ns	-
	Height (cm)	45.3 ± 10.1	44	34.1 ± 11.3	44	*t*_43_ = 4.5	< 0.05	N
	Max width (cm)	129.4 ± 50.3	44	95.2 ± 51.7	44	*t*_43_ = 3.6	< 0.05	N
*Lepidobolus deserti*	Stage	Cat.	16	Cat.	16	χ ^2^ = 2.9, *d.f.* = 15	ns	-
	Height (cm)	41.9 ± 10.4	16	43.1 ± 9.7	16	*t*_15_ = −0.1	ns	-
	Max width (cm)	93.3 ± 16.1	16	57.5 ± 7.6	16	*t*_15_ = 3.6	< 0.05	N
Log	Length (cm)	250.1 ± 103.0	6	263.5 ± 182.9	18	*t*_20_ = −0.3	ns	N
	Width (cm)	20.3 ± 5.5	6	19.6 ± 8.4	18	*t*_20_ = 0.4	ns	
	Entrance diameter (cm)	7.0 ± 3.6	6	11.0 ± 8.2	18	*t*_17_ = −2.1	< 0.05	N
*Schoenus hexandrus*	Stage	“Stage 5”	1	NA				
	Height (cm)	24.3	1					
	Width (cm)	68.5	1					
Mallee stump	Width (cm)	20.5	1					
	Height aboveground (cm)	100.1	1					
	Entrance diameter (cm)	18.7	1					
	Depth (cm)	20.1	1					
Bark	Length (cm)	97.5	1					
	Width (cm)	10.2	1					
	Entrance diameter (cm)	4.5	1					

The GLMM variables that best explained differences between shelter and random plots are given in Table 3. Prior to fitting models, we determined that Ground and Middle were correlated (|*r*| = 0.68). Ground was removed from further models to avoid multicollinearity. The variable of Lower had a large and significant impact on shelter selection (Table 3). Overall, 12 models performed well (Δ*i* ≤ 2) at explaining differences between the shelter and random plots of *S. psammophila*. The best GLMMs are given in Table 4. All of the best models included the variable of Lower, indicating that cover in the lower stratum habitat is an important determinate of the location of the shelters of *S. psammophila*.

**Table 3.** List of habitat variables from the best generalized linear mixed-effects models (GLMMs) at the plot level including effect size, *SE*, *Z*-statistic, and *P*-value.

Model variable	Effect size	± *SE*	*Z*-statistic	*P-*value
Dist.Crest	−0.19	0.12	1.5	0.13
Elevation	0.09	0.11	0.9	0.37
Floristic richness	−0.18	0.11	1.7	0.09
Lower	1.00	0.13	7.7	< 0.001
Middle	0.13	0.12	1.1	0.25
Minimum fire age	−0.09	0.13	0.9	0.37
Terrain slope	0.22	0.12	1.9	0.06

**Table 4.** The most parsimonious (Δ*i* ≤ 2) and best-fitting generalized linear mixed-effects models (GLMMs) used to explain differences between shelter and random plots for *Sminthopsis psammophila*. *K* = the number of estimated parameters, AICc = Akaike’s information criterion for small samples, Δ*i* = the difference in AICc score compared to the most parsimonious model, ω*i* = Akaike weights, and pseudo-*R*^2^ = the proportion of residual deviance explained by the model. Final models have been averaged.

Model	*K*	AICc	Δ*i*	ω*i*	Pseudo-*R*^2^
Lower + floristic richness + terrain slope	6	509.4	0	0.138	0.21
Lower + dist.Crest + floristic richness + terrain slope	7	509.7	0.30	0.119	0.22
Lower + terrain slope	5	510.2	0.76	0.095	0.21
Lower + floristic richness + Middle + terrain slope	7	510.3	0.85	0.090	0.22
Lower + dist.Crest + terrain slope	6	510.4	0.99	0.084	0.21
Lower + dist.Crest + floristic richness + Middle + terrain slope	8	510.4	0.99	0.084	0.22
Lower + dist.Crest + floristic richness	6	510.6	1.13	0.079	0.21
Lower + dist.Crest	5	510.8	1.38	0.069	0.20
Lower + elevation + floristic richness + terrain slope	7	510.9	1.44	0.067	0.22
Lower + dist.Crest + elevation + floristic richness + terrain slope	8	511.1	1.68	0.060	0.22
Lower + dist.Crest + floristic richness + minimum fire age + terrain slope	8	511.2	1.74	0.058	0.22
Lower + dist.Crest + Middle floristic richness	7	511.2	1.75	0.058	0.22

**Fig. 5.**
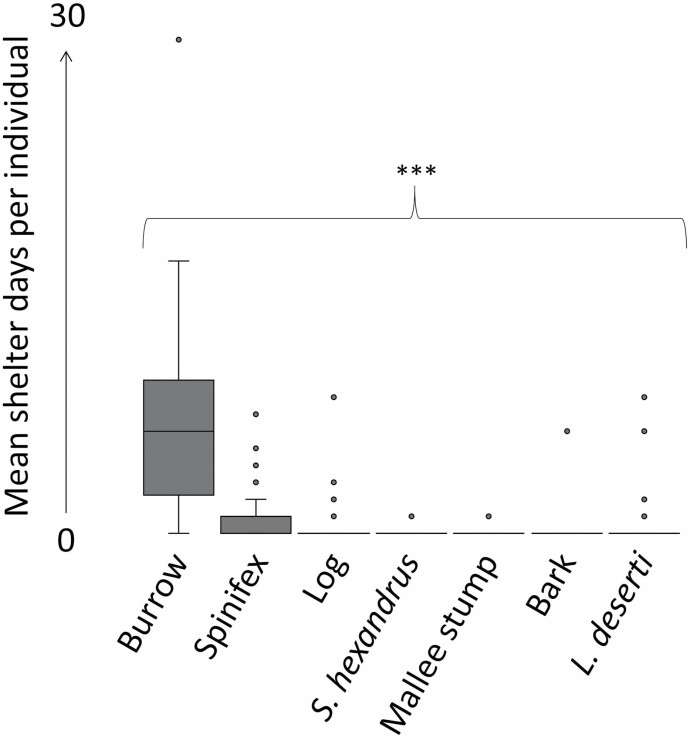
The mean ± *SE* number of shelter days (the number of days each shelter type was used) by *Sminthopsis psammophila* (*n* = 40) in the Western Australian Great Victoria Desert. Significant differences are indicated by *** (*P* < 0.001).

*Sminthopsis psammophila* preferred to shelter in burrows (Table 5). Burrows were selected on 278 shelter days (Table 5). There was a significant effect of shelter type on mean shelter days (one-way ANOVA, *F*_6,252_ = 39.7, *P* < 0.001; Table 5; Fig. 5). The number of burrow shelter days was greater than that of all other shelter days (Tukey’s test, *P* < 0.001 for all shelter type comparisons). Most burrows (*n* = 130) were concealed under mature (“Stage 3” to “Stage 5”) spinifex hummocks (Fig. 2). A few burrows were under no vegetation (*n* = 8) or under shrubs only (*n* = 3; Supplementary Data SD5). We measured 108 unoccupied burrows. Entrances of used burrows were significantly smaller than those of random burrows, but burrow depths were not significantly different (Table 2). Burrow entrance aspects were significantly different from a random distribution (north = 51, south = 34, west = 15, east = 8; Table 2). Individuals were observed excavating burrows on multiple occasions. Burrows were easily identifiable as those of *S. psammophila* due to their dimensions and location, and fresh spoil heaps were situated at most burrow entrances. One female was recorded on video taking nesting material into a maternity burrow. We observed several individuals sun basking (passive rewarming) at burrow entrances, particularly during cooler conditions.

**Table 5.** Shelter selection of *Sminthopsis psammophila* grouped by sex and reproductive status. Sum totals are given in the top row for each category and mean ± *SD* in italics.

Group (*n* individuals tracked)	Shelters	Shelter days	Days before moving	Distance between shelters (m)	dist.Crest (m)	Burrow shelter days	Spinifex (*Triodia* spp.) shelter days	*Lepidobolus deserti* shelter days	*Schoenus hexandru s* shelter days	Log shelter days	Bark shelter days	Mallee stump shelter days
Reproductive male (RM; *n* = 15)	51	106	-	-	-	94	5	2	0	5	0	0
	*3 ± 2*	*7 ± 5*	*3 ± 2*	*212 ± 239*	*85 ± 79*	*6 ± 4*	*0 ± 1*	*0 ± 1*	*0 ± 0*	*0 ± 1*	*0 ± 0*	*0 ± 0*
Nonreproductive male (nRM; *n* = 10)	74	96	-	-	-	62	18	14	0	1	0	1
	*7 ± 2*	*10 ± 4*	*1 ± 0*	*127 ± 44*	*173 ± 275*	*6 ± 4*	*2 ± 2*	*1 ± 3*	*0 ± 0*	*0 ± 0*	*0 ± 0*	*0 ± 0*
Reproductive female (RF; *n* = 7)	36	83	-	-	-	61	7	1	0	8	6	0
	*5 ± 4*	*12 ± 17*	*1 ± 1*	*120 ± 69*	*392 ± 357*	*9 ± 10*	*1 ± 2*	*0 ± 0*	*0 ± 0*	*1 ± 3*	*1 ± 2*	*0 ± 0*
Nonreproductive female (nRF; *n* = 8)	50	78	-	-	-	61	16	0	1	0	0	0
	*6 ± 4*	*10 ± 4*	*1 ± 0*	*100 ± 39*	*263 ± 333*	*8 ± 5*	*2 ± 3*	*0 ± 0*	*0 ± 0*	*0 ± 0*	*0 ± 0*	*0 ± 0*
*S. psammophila* (*n* = 40)	211	363	-	-	-	278	46	17	1	14	6	1
	*5 ± 3*	*9 ± 8*	*2 ± 2*	*154 ± 159*	*205 ± 287*	*7 ± 6*	*1 ± 2*	*0 ± 2*	*0 ± 0*	*0 ± 1*	*0 ± 1*	*0 ± 0*

Forty-five spinifex hummocks (*T. desertorum, n* = 29; *T.* sp. *rigidissima*, *n* = 11; *T. basedowii*, *n* = 5), 16 *L. deserti* hummocks, and one *S. hexandrus* hummock were used as shelters (Table 5; Supplementary Data SD5). Most (55%) spinifex hummock shelters were mature (“Stage 4” to “Stage 5”) and “Stage 1” and “Stage 2” hummocks were not used (Fig. 2). We measured 44 spinifex shelters, 16 *L. deserti* shelters, and the *S. hexandrus* hummock. Spinifex shelters were significantly wider and taller than random spinifex hummocks (Table 2). All *L. deserti* shelters were “Stage 4” hummocks and were significantly more mature and wider, but not taller, than random *L. deserti* hummocks (Table 2) and most *L. deserti* shelters had shallow central scrapes < 5 cm deep.

Log shelters (*n* = 6) were used for 14 shelter days (Table 5; Supplementary Data SD5). Log shelter lengths and widths were not significantly different from those of random logs, but log entrance diameters were significantly smaller than random log entrances (Table 2). One log shelter in a *E. youngiana* was 0.8 m aboveground. All other log shelters were at ground level. The bark shelter was used for six shelter days and a hollow in a mallee, *Eucalyptus* sp., stump in “Burned” habitat was used for one shelter day (Tables 2 and 5; Supplementary Data SD5).

At the plot level, a two-way ANOVA showed a significant main effect of sex on dist.Crest (M = 133 ± 213 m; *F* = 328 ± 359 m; *F*_2,208_ = 25.3, *P* < 0.001) but no main effect of reproductive status on dist.Crest (R = 205 ± 275; nR = 205 ± 298; *F*_2,208_ = 0.04, *P* > 0.05; Table 5). The interaction effect was significant (*F*_2,208_ = 8.1, *P* < 0.001). RM shelters had a significantly smaller dist.Crest compared with RF shelters (Tukey test, *P* < 0.001) and nRF shelters (Tukey’s test, *P* < 0.01) but not nRM shelters (Tukey’s test nonsignificant). nRM shelters had a significantly smaller dist.Crest compared with RF shelters (Tukey’s test, *P* < 0.001) but there were no differences between the remaining groups. There was a significant effect of sex on terrain slope (M = 4.7 ± 6.1°, F = 2.4 ± 3.1°; *W* = 3,803, *P* < 0.01) but there were no other significant effects of sex or reproductive status on shelter variables at the plot level.

At the shelter level, RM had significantly deeper burrows (53 ± 47 cm) than all other groups (nRM = 32 ± 50 cm, RF = 34 ± 28 cm, nRF = 31 ± 26 cm; Kruskal–Wallis χ ^2^ = 11.1, *d.f.* = 3, *P* < 0.05; pairwise Mann–Whitney test, *P* < 0.001 for RM and all other groups). Burrow height and width did not differ among groups (Kruskal–Wallis tests were not significant). RM remained in the same shelter for a significantly higher number of days before moving to a new shelter compared with all other groups (Kruskal–Wallis χ ^2^ = 10.3, *d.f.* = 3, *P* < 0.05; pairwise Mann–Whitney test, *P* < 0.001 for RM and all other groups). RM had significantly fewer shelters than all other groups (Kruskal–Wallis = 11.9, *d.f.* = 3, *P* < 0.05; pairwise Mann–Whitney test, *P* < 0.001 for RM and all other groups; Table 5).

**Fig. 6.**
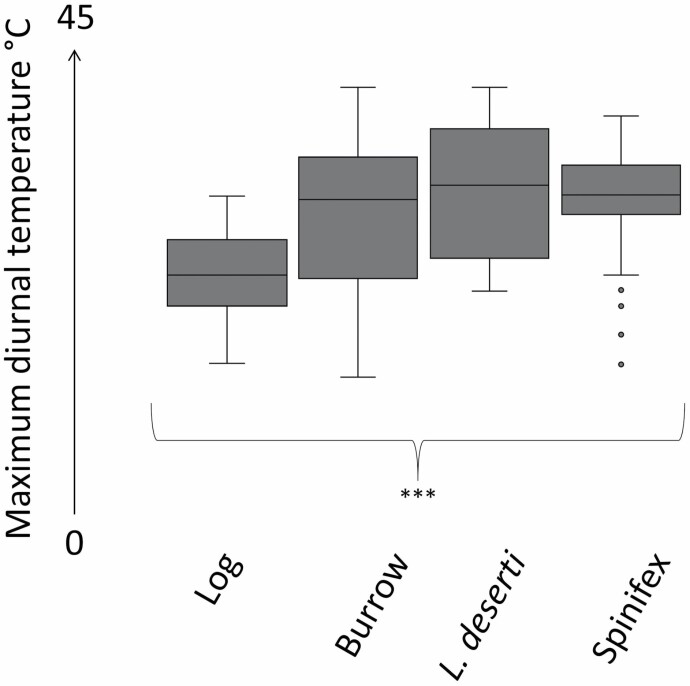
Mean maximum ambient temperatures (*T*_A_ °C) were measured by the Tropicana Gold Mine (TGM) weather station. Logs were selected by *Sminthopsis psammophila* on significantly cooler days compared with all other measured shelter types, indicated by *** (*P* < 0.001).

iButtons recorded *T*_r_ in six burrows, five spinifex hummocks, five *L. deserti* hummocks, three logs, a *S. hexandrus* hummock, and a mallee stump hollow for a mean of 56 ± 33 days per shelter. GT_max_ was 61°C on 29 November 2015 at 1300 h (ground temperatures can become very high due to reflective heat) and GT_min_ was −4.5°C on 7 June 2017 at 0500 h. Burrow *T*_r_ was significantly lower than GT_r_ (*t*_5_ = −7.3, *P* < 0.001) and *L. deserti T*_r_ was significantly lower than GT_r_ (*t*_4_ = −4.0, *P* < 0.05) but no significant thermal effects were detected for spinifex or log shelters (Table 6). Mean T °C in two log shelters was 10°C warmer than GT. There was a significant effect between shelter type and *T*_max_ (Kruskal–Wallis = 17.3, *d.f.* = 6, *P* < 0.05; pairwise Mann–Whitney test, *P* < 0.05 for logs and all other shelter types) and log shelters were selected on cooler days (Fig. 6). There was a significant effect between habitat class and HT_x_ (one-way ANOVA, *F*_4,1134_ = 7.4, *P* < 0.001; Table 7). “Swale or sand plain” and “North slope” habitat classes were significantly warmer than “Mulga” and “South slope” habitats (Tukey’s tests, *P* < 0.01 for all comparisons). “Mulga” habitats were significantly cooler than “Crest” habitats (*P* < 0.05) but there were no other significant differences of HT_x_.

**Table 6.** Thermochron iButton data logger temperature recordings made within the diurnal shelters of *Sminthopsis psammophila*. T = temperature (°C) is given as mean *± SD*; min. = minimum, max. = maximum.

Shelter type	*n* iButtons (replicates)	Mean days recorded	Mean max. shelter T (°C)	Mean min. shelter T (°C)	Mean shelter T (°C) range	Mean max. control T (°C)	Mean min. control T (°C)	Mean control T (°C) range	*P-*value (paired *t-*test)
Burrow	6	33	31 ± 8	15 ± 5	16 ± 10	46 ± 15	5 ± 5	40 ± 13	< 0.001
*Triodia* spp.	5	35	42 ± 12	2 ± 5	40 ± 10	47 ± 15	3 ± 5	43 ± 13	ns
*Lepidobolus deserti*	5	26	36 ± 7	7 ± 6	29 ± 7	49 ± 12	2 ± 6	47 ± 9	< 0.05
Log	3	39	46 ± 7	2 ± 5	37 ± 3	43 ± 17	7 ± 5	36 ± 14	ns
*Schoenus hexandrus*	1	83	44	11	33	52	11	41	NA
Mallee stump	1	85	44	−0.5	45	52	−1	52	NA
Mean (all shelters^a^)	4 ± 2	56 ± 33	38 ± 10	8 ± 7	30 ± 12	48 ± 4	5 ± 4	43 ± 12	< 0.05

^a ^Bark shelter temperatures were not measured as the shelter was occupied.

**Table 7.** Thermochron iButton data logger temperature recordings made within the habitat classes of *Sminthopsis psammophila*. T = temperature (°C) is given as mean *± SD*.

iButton replicate	Dates deployed	*n* days deployed	Swale and sand plain T (°C)	Crest T (°C)	North slope T (°C)	South slope T (°C)	Mulga T (°C)	Warmest habitat	Coolest habitat
1	8 March 2017 to 20 March 2017	20	27 ± 11	26 ± 10	27 ± 11	25 ± 8	24 ± 9	Swale and sand plain and North slope	Mulga
2	8 March 2017 to 10 April 2017	33	25 ± 12	24 ± 10	24 ± 11	19 ± 8	19 ± 10	Swale and sand plain	South slope and Mulga
3	10 April 2017 to 4 July 2017	85	15 ± 7	17 ± 10	16 ± 13	14 ± 9	14 ± 12	Crest	South slope and Mulga
4	23 March 2018 to 16 June 2018	84	19 ± 9	18 ± 8	19 ± 10	17 ± 8	18 ± 9	Swale and sand plain	South slope
5	10 September 2018 to 29 September 2019	19	28 ± 13	28 ± 9	30 ± 12	27 ± 10	26 ± 14	North slope	Mulga
6	14 October 2018 to 13 November 2018	29	26 ± 12	26 ± 10	29 ± 14	27 ± 13	25 ± 11	North slope	Mulga

## Discussion

Shelter provides protection from climate extremes, predation, and wildfires, and influences morphological, behavioral, and physiological adaptations in arid-dwelling species (Körtner et al. 2008; Pavey and Geiser 2008; Degen 2012). Consequently, shelter requirements dictate the range and occurrence of many species. Prior to our study, studies of sheltering preference for *S. psammophila* were limited to outlying populations with inconsistent results (Churchill 2001a, 2001b; McLean 2015; Moseby et al. 2016). However, the use of burrows for micro-refuges, torpor, and passive rewarming is common for desert fauna (e.g., Kinlaw 1999; Lovegrove et al. 1999; Warnecke et al. 2008; Degen 2012). In our study, nearly 50% of burrow entrances faced north and shelters were selected within warmer habitat classes facilitating passive rewarming and reducing energetic costs by lowering the need for an increase in metabolic rate (Warnecke et al. 2008; Degen et al. 2012). The importance of burrows for *S. psammophila* may have been overlooked as many *Sminthopsis* spp. are reported to inhabit other types of subterranean shelter while few are reported to excavate their own burrows (Haythornthwaite and Dickman 2006; Waudby and Petit 2017; Baker and Dickman 2018).

Dune “Crest” habitats were rarely used by *S. psammophila* during diurnal sheltering, despite the species’ common name of the “sandhill” dunnart. Instead, *S. psammophila* preferred to shelter within swales, sand plains, and dune slopes with a dense lower habitat stratum and vegetation of mature seral stages. “Crest” habitats may be used infrequently due to their finer soil substrates that are inadequate for stable burrow construction (*S. psammophila* was observed excavating burrows and usually did not usurp those of other species). As temperature fluctuations are smaller in harder soils than in wind-blown, fine-grained soils, this is an important adaptation of *S. psammophila* shared by many burrowing desert mammals (Bennett et al. 1988). In addition, “Crest” habitats lack a dense lower stratum and are popular elevated hunting routes within the otherwise low-lying habitats of the WAGVD. The habitats preferred by *S. psammophila* develop in the absence of wildfire and contain the range and abundance of sites required for shelter. Consequently, the conservation of long-unburned habitats is vital for *S. psammophila* (and other sympatric taxa) in the arid zone (Masters 1993; Letnic and Dickman 2005; Letnic and Dickman 2010; Moseby *et al.* 2016). The preferred habitat age (32+ years postfire) for supporting *S. psammophila* shelters is likely underestimated as our analyses were limited by the age (40 years) of WAGVD satellite imagery. Improved methods for assessing fire age for the WAGVD are in progress, including *Callitris* sp. dendrochronology (O’Donnell et al. 2010).

The importance of burrows for *S. psammophila* may also be obscured by a reported sheltering preference for “Stage 3” spinifex hummocks on Eyre Peninsula (EP—Churchill 2001a). While we occasionally recorded spinifex hummocks as shelters, they were used infrequently and were not thermally advantageous. The extreme ground temperature ranges recorded in our WAGVD study (−4.5 to 61°C) are not physiologically survivable for *S. psammophila* without burrowing or the use of thermally insulative shelters (Withers and Cooper 2009). Further, burrow use is an essential arid zone adaptation to survive passing wildfire fronts (Friend 1993; Long 2009). When we combine our data set with Churchill’s (2001b) unpublished data set we conclude that 62% of all shelters recorded for *S. psammophila* using radiotracking have been burrows.

Climatic or ecological differences may affect shelter selection across populations of *S. psammophila* (Churchill 2001a). For example, annual temperatures in WAGVD and Yellabinna Regional Reserve (YRR) habitats are more extreme (Supplementary Data SD3), and WAGVD and YRR habitats are long-unburned and dominated by *T. desertorum*. In contrast, EP habitats are generally milder (Supplementary Data SD3), more recently burned, and dominated by *T. irritans* and *T. basedowii*, which are usually faster-growing, dome-forming spinifex species (Churchill 2001b). As the three populations are estimated to have been isolated for many thousands of years, it is possible that *S. psammophila* exhibits differing shelter behaviors across populations (McLean *et al.* 2019). Thus, site-specific habitat characteristics (particularly *Triodia* spp. and fire age) should be considered during the survey and conservation of *S. psammophila*. We suggest further research on this phenomenon using radiotracking across different EP populations.

*Lepidobolus deserti* and *S. hexandrus* are ecologically intriguing shelter choices as their soft foliage likely provides reduced protection from predation than the ubiquitous sharp-leaved spinifex hummocks that are more commonly used by sympatric arid zone mammals (Dawson and Bennett 1978; Burbidge *et al.* 1988; Bos *et al.* 2002). However, shallow scrapes within *L. deserti* shelters were thermally beneficial, indicating this may be a more important survival response for *S. psammophila*. Logs were important shelters for *S. psammophila* in mild conditions and are opportunistically used by other dunnart species such as *S. dolichura* and *S. crassicaudata* (Morton 1978; Friend and Pearson 1995; Churchill 2001b). In our study, log shelters were usually warmer and selected on cooler days, potentially to aid passive rewarming (Fig. 6). Diurnal movements between shelters were observed on three occasions, but were not common and individuals moved less than 25 m. The range of shelter sites selected by *S. psammophila*, which included two semiarboreal shelters (the burned mallee stump and a high log), also indicate an opportunistic strategy by some individuals that permits a wider use of the landscape as in a smaller dasyurid, the common planigale, *P. maculata*, which uses tree holes as well as soil cracks as shelters (Baker and Dickman 2018).

*Sminthopsis psammophila* shares common sheltering preferences and behavioral adaptations with many species of small desert mammal worldwide (McNab and Morrison 1963; Downs and Perrin 1990; Kinlaw 1999; Degen 2012). However, *S. psammophila* displays some unique sheltering characteristics among the Australian dasyurids. For example, *S. psammophila* often constructs its own burrows. Reproductively active males invest in superior (deep) burrows and are probably territorial, as has been observed in captivity (Lambert et al. 2011). This may be related to the proximity of receptive females during the reproductive season. The construction of deep burrows is energetically expensive, and their repeated use can increase predation risk. However, their constant microclimates are physiologically beneficial and are therefore worth retaining, especially for reproductively active males that have higher energetic costs due to moving long distances (J. Riley, pers. obs.). In addition, *S. psammophila* often reuses shelter sites, particularly burrows (*n* = 40 reused over 175 shelter days), reflecting either the paucity of suitable shelter sites across the landscape, or a benefit of burrow reuse, i.e., the physiological benefits of repeated burrow use outweigh the increased predation risk or an increased buildup of parasites (Baker and Dickman 2018). On average, five *S. psammophila* shelters were spaced over a mean home range of 70 ha (J. Riley, pers. obs.). Conversely, the threatened Julia Creek dunnart, *S. douglasi*, has much smaller home ranges (0.05–0.40 ha—Woolley 2017; 0.5–8.0 ha—Mifsud 1999), utilizes cracks or holes in clay soils, and displays a reduced fidelity to shelter sites. Similarly, two small planigale species (*P. gilesi* and *P. tenuirostris*) and several other dunnart species (*S. crassicaudata*, *S. dolichura*, *S. macroura*, and *S. youngsoni*) show little shelter fidelity and are highly mobile (Morton 1978; Read 1987; Dickman et al. 1995; Haythornthwaite and Dickman 2006; Waudby and Petit 2017). Such strategies have been linked to survival in unpredictable, resource-poor arid environments. However, the apparently sedentary life strategy of some *S. psammophila* individuals infers that there are benefits to staying in one location, such as retaining information on the location of resources such as mates or stable food patches, rather than drifting toward opportunistic or sparse resources in unknown locations. Stable home ranges are common for many small arid zone mammals globally, for example, elephant shrews (Macroscelididae) in the Kalahari Desert have such fidelity to their ranges that their runways are etched into the topsoil and regularly cleaned to facilitate easy movement (Baker and Dickman 2018). While some *S. psammophila* were sedentary, not all individuals reused burrows and some individuals were highly mobile. Therefore, we agree that *S. psammophila* displays both resident and transient sheltering behavior as proposed by McLean *et al.* (2019).

Catastrophic wildfires and habitat loss are immediate concerns for *S. psammophila* (Woinarski and Burbidge 2016). The ecologically sustainable management of wildfires is central to Australian desert conservation and the use of cultural or traditional burning is well supported for wildfire management (Bowman 1995; Brennan et al. 2012; Moorcroft et al. 2012). Consultations with local indigenous experts are recommended for the correct timing, location, and scale of preventative burns. Emerging conservation methods that may be useful include deploying artificial lower stratum structures in key habitats (Bleicher and Dickman 2020) and controlling feral mesopredators in recently burned habitats (McGregor et al. 2015, 2017). The tendency of *S. psammophila* to occupy shallow burrows, while currently sufficient for the species’ survival on the southern fringe of the arid zone, may predispose a vulnerability to anthropogenic climate change. Hence, this is an important area for further study. The specific habitat requirements of *S. psammophila* influence the species’ range, which is largely restricted to the southern, temperate-influenced margins of the GVD. Thus, the management of *S. psammophila* must consider these preferences for the effective conservation of the species now and in the future.

## Supplementary Material

gyab024_suppl_Supplementary_Data_1

gyab024_suppl_Supplementary_Data_2

gyab024_suppl_Supplementary_Data_3

gyab024_suppl_Supplementary_Data_4

gyab024_suppl_Supplementary_Data_5

## Supplementary Data

**Supplementary Data SD1.**—Site locations and descriptions for radiotracked and GPS-tracked *Sminthopsis psammophila* in the Western Australian Great Victoria Desert (WAGVD) between 2015 and 2019. Habitat classes: 1) “Swale or sand plain”; 2) “Crest”; 3) “North slope”; 4) “South slope”; 5) “Woodland”; 6) “Mulga”; or 7) “Burned.” Detailed habitat class descriptions are given in Table 1. WGS 1984 datum. * = site partially burned.

**Supplementary Data SD2.**—Mean, maximum (Max), and minimum (Min) annual temperatures (°C) recorded by the Tropicana Gold Mine (TGM) weather station during the tracking study period (top). Total annual rainfall (mm) and standardized sandhill dunnart (SHD) captures (per 100 trap-nights) during the study period (bottom).

**Supplementary Data SD3.**—Long-term weather data for the Western Australian Great Victoria Desert (WAGVD) study site, Eyre Peninsula (EP), and Yellabinna Regional Reserve (YRR) *Sminthopsis psammophila* population sites. Data were downloaded from the Bureau of Meteorology (BOM 2020).

**Supplementary Data SD4.**—Bonferroni’s confidence intervals were calculated to establish positive, negative, or absent habitat class selection for the shelters of *Sminthopsis psammophila.* Burned habitat classes were excluded from analysis.

**Supplementary Data SD5.**—(a) Exposed burrow with emerging *Sminthopsis psammophila* individual inset; (b) Concealed burrow under a “Stage 5” *Triodia desertorum* hummock; (c) “Stage 3” *T. desertorum* hummock; (d) *Lepidobolus deserti* hummock with individual inset; (e) *Schoenus hexandrus* hummock with individual inset; (f) log shelter; (g) bark shelter; (h) burned mallee stump shelter with individual inset.
