# Supplementary material for: Diurnal sheltering preferences and associated conservation management for the endangered sandhill dunnart, Sminthopsis psammophila
Source: J Mammal. 2021 Apr 23;102(2):588–602. doi: 10.1093/jmammal/gyab024 (PMC8245887; doi:10.1093/jmammal/gyab024)
Supplement: gyab024_suppl_Supplementary_Data_1 [file gyab024_suppl_supplementary_data_1.docx]

| Site | Longitude | Latitude | Available habitat classes | Date of most recent burn at time of tracking | Dominant spinifex *Triodia* spp. | Dominant shrub  species | Mean minimum fire age (years) | Minimum fire age range (years) | *S. psammophila* ID |
| --- | --- | --- | --- | --- | --- | --- | --- | --- | --- |
| 7 | 124.2499 | -29.2568 | 1 – 6 | Dec 2001 | *T. desertorum* | *Thryptomene biseriata, Aluta maisonneuvei, Acacia spinosissima* | 30+ | 12 - 40+ | 08, 12, 15, 16, 32, 37, 41 |
| 9 | 124.2866 | -29.2582 | 1 – 6 | N/A | *T. desertorum* | *A. maisonneuvei, T. Biseriata, A. Spinosissima* | 40+ | 40+ | 05, 26, 40 |
| 10 | 124.3052 | -29.2567 | 1 – 7 | N/A  * Dec 2016 | *T. desertorum* | *A. maisonneuvei, Baeckea* sp. *GVD, Allocasuarina spp.* | 39+ | 40+  0 - 40+ | 03, 04, 09  * 21, 22, 30, 43 |
| 11 | 124.0636 | -29.5568 | 1 – 6 | Nov 2002 | *T. desertorum* | *Bertya dimerostigma,* *A. Maisonneuvei, Hakea francisiana, Grevillea juncifolia* | 12.5 | 10 - 20 | 01, 10 |
| 12 | 124.0923 | -29.2544 | 1 - 6 | N/A | *T. desertorum and T. basedowii* | *Acacia helmsiana, A. maisonneuvei, G. juncifolia, Baeckea* sp. *GVD* | 40+ | 40+ | 35 |
| 13 | 124.1201 | -29.2545 | 1 – 6 | N/A | *T. desertorum and T. basedowii* | *Acacia helmsiana, A. maisonneuvei, G. juncifolia, B. dimerostigma* | 40+ | 40+ | 14, 29, 39 |
| 14 | 124.3823 | -29.2507 | 1, 5, 6 | January 2002 | *T. basedowii and T.* sp. *rigidissima* | *A. maisonneuvei, Acacia incurvaneura and Acacia caesaneura* | 14 | 12 - 40+ | 02, 06, 17, 23 |
| 15 | 124.2703 | -29.2568 | 1 - 6 | N/A | *T. desertorum* | *A. maisonneuvei, Leptospermum* sp., *G. didymobotrya* | 40+ | 40+ | 07, 11, 18, 27, 31, 36 |
| 20 | 123.9806 | -29.2432 | 1 - 5 | N/A | *T. desertorum and T. basedowii* | *T. biseriata, A. maisonneuvei* | 38+ | 10 - 40+ | 13, 33, 34 |
| 21 | 124.4437 | -29.2491 | 1 - 6 | January 2004 | *T. desertorum* | *T. biseriata, A. maisonneuvei, Dodonaea viscosa* | 32+ | 13 - 40+ | 19, 24 |
| 22 | 124.4340 | -29.2473 | 1 - 6 | N/A | *T. desertorum* | *Baeckea* sp. *GVD, A. maisonneuvei* | 40+ | 40+ | 42 |
| 23 | 124.1809 | -29.2558 | 1 - 6 | N/A | *T. basedowii and T.* sp. *rigidissima* | *A. desertorum, A. jamesiana* | 17.5 | 15 - 20 | 38 |
