# Supplementary figures and images for: Diurnal sheltering preferences and associated conservation management for the endangered sandhill dunnart, Sminthopsis psammophila
Source: J Mammal. 2021 Apr 23;102(2):588–602. doi: 10.1093/jmammal/gyab024 (PMC8245887; doi:10.1093/jmammal/gyab024)

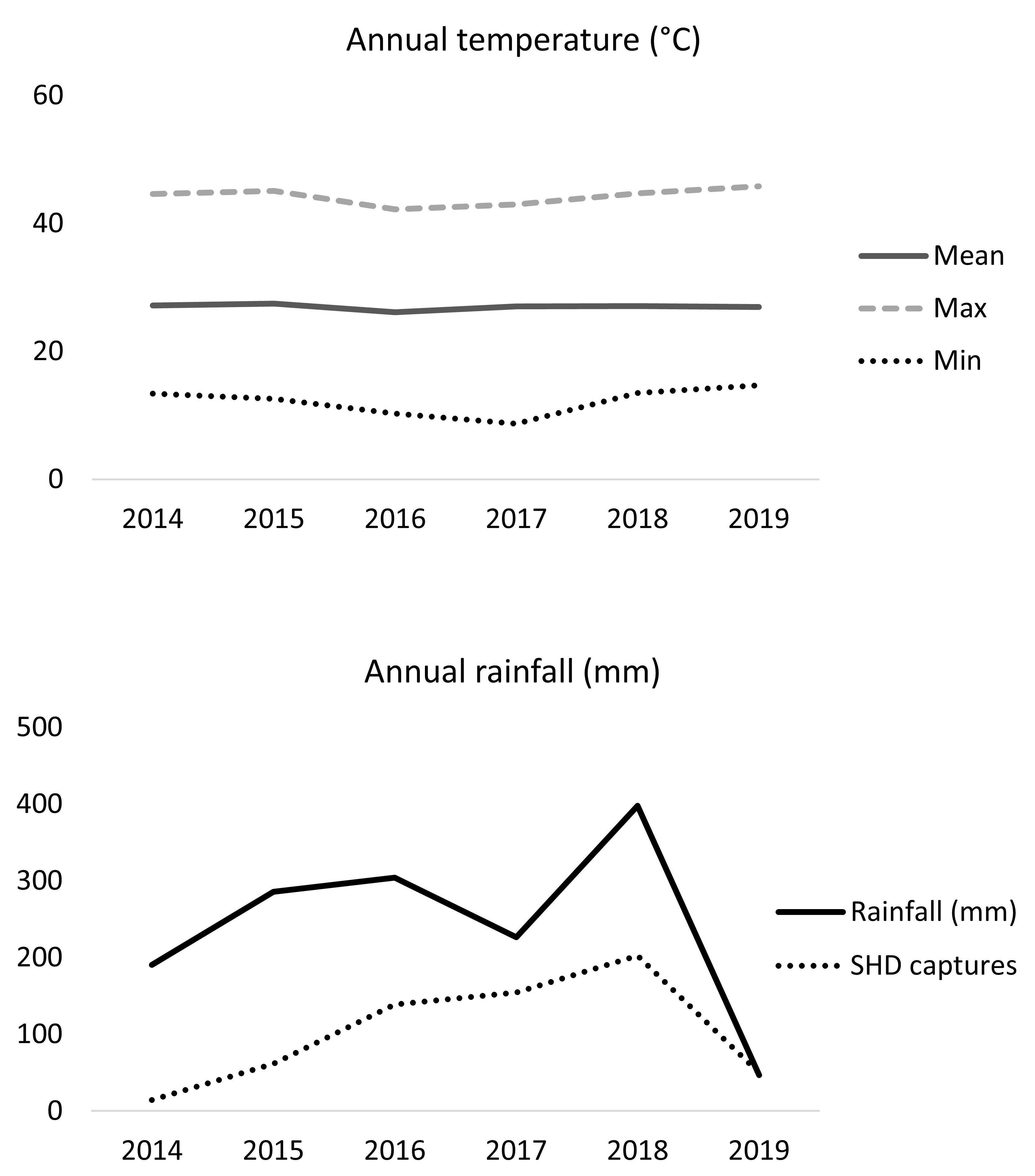

Supplement: gyab024_suppl_Supplementary_Data_2 [file gyab024_suppl_supplementary_data_2.jpeg]

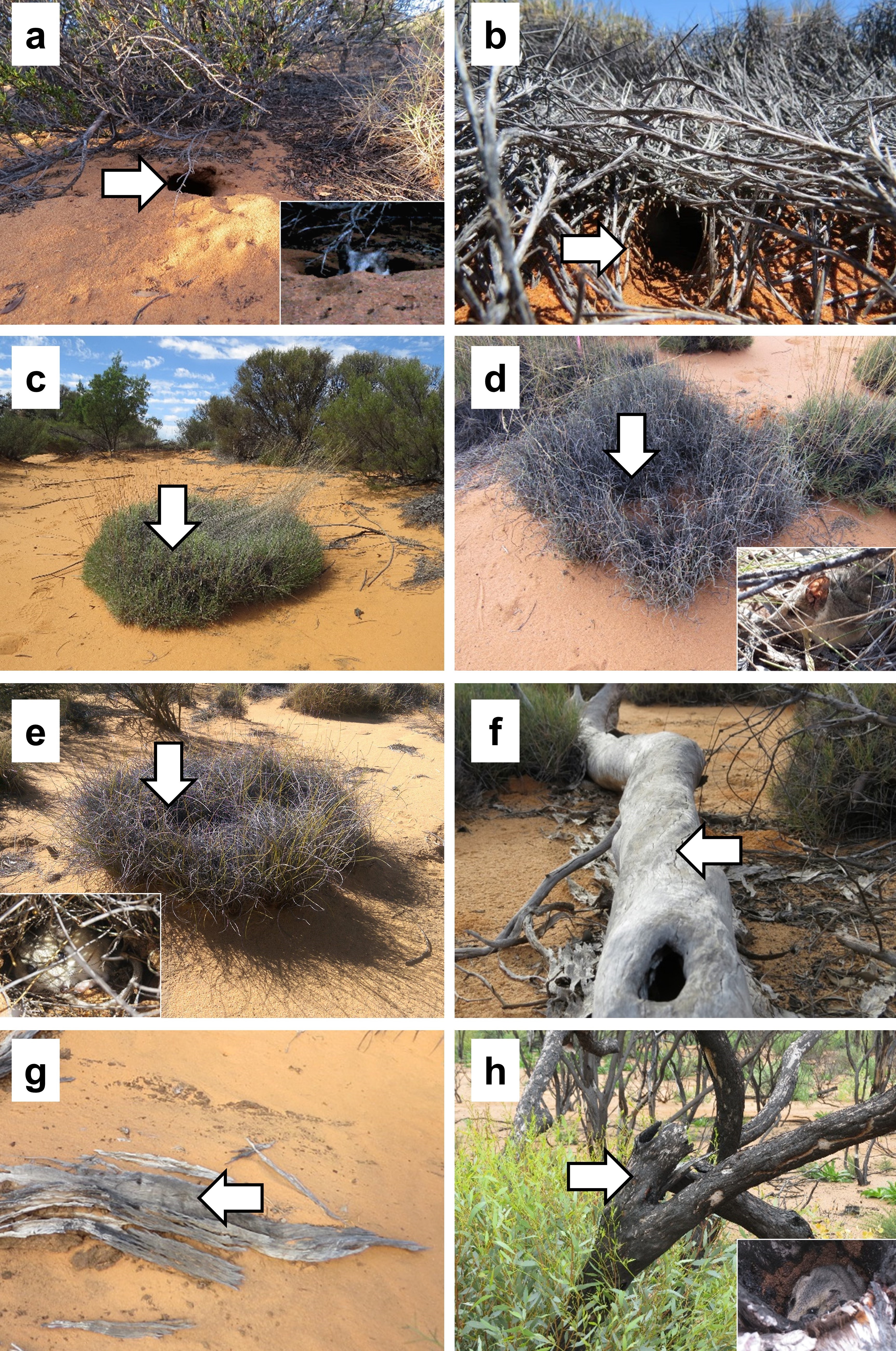

Supplement: gyab024_suppl_Supplementary_Data_5 [file gyab024_suppl_supplementary_data_5.jpeg]
