# Supplementary material for: Diurnal sheltering preferences and associated conservation management for the endangered sandhill dunnart, Sminthopsis psammophila
Source: J Mammal. 2021 Apr 23;102(2):588–602. doi: 10.1093/jmammal/gyab024 (PMC8245887; doi:10.1093/jmammal/gyab024)
Supplement: gyab024_suppl_Supplementary_Data_3 [file gyab024_suppl_supplementary_data_3.docx]

| Population | Weather station | Years measured | T_LT_.max (ºC) | T_LT_.min (ºC) | T_LT_.hi  (ºC) | T_LT_.lo  (ºC) |
| --- | --- | --- | --- | --- | --- | --- |
| Study site (WAGVD) | Laverton Aero 012305 | 1991 - 2020 | 27.2 | 14.1 | 47.6 | -3.3 |
| Eyre Peninsula (EP) | Kimba 018040 | 1997 - 2020 | 23.6 | 10.3 | 47 | -1.7 |
| Yellabinna Regional Reserve (YRR) | Tarcoola Aero 016098 | 1997 - 2020 | 27.7 | 12.1 | 49.1 | -3.8 |
