# Supplementary material for: Diurnal sheltering preferences and associated conservation management for the endangered sandhill dunnart, Sminthopsis psammophila
Source: J Mammal. 2021 Apr 23;102(2):588–602. doi: 10.1093/jmammal/gyab024 (PMC8245887; doi:10.1093/jmammal/gyab024)
Supplement: gyab024_suppl_Supplementary_Data_4 [file gyab024_suppl_supplementary_data_4.docx]

| Habitat | Area (ha) | Number of shelters | Proportion of use expected | Proportion of use observed | Chi square value | Selection |
| --- | --- | --- | --- | --- | --- | --- |
| Swale | 333.5 | 88 | 0.21 | 0.42 | 44.95 | Positive |
| North slope | 155.5 | 46 | 0.10 | 0.22 | 31.60 | Positive |
| South slope | 141.1 | 39 | 0.09 | 0.19 | 22.14 | Positive |
| Woodland | 497.3 | 21 | 0.31 | 0.10 | 30.15 | Negative |
| Crest | 58.7 | 12 | 0.04 | 0.06 | 1.97 | Absent |
| Mulga | 429.2 | 4 | 0.27 | 0.02 | 49.02 | Negative |
| Totals | **1615.3** | **210** | **1.00** | **1.00** | **179.83** | - |
